# Environmental Quality, Developmental Plasticity and the Thrifty Phenotype: A Review of Evolutionary Models

**Published:** 2007-06-19

**Authors:** Jonathan CK Wells

**Affiliations:** Reader in pediatric Nutrition

**Keywords:** thrifty phenotype, developmental plasticity, adaptation

## Abstract

The concept of the thrifty phenotype, first proposed by Hales and Barker, is now widely used in medical research, often in contrast to the thrifty genotype model, to interpret associations between early-life experience and adult health status. Several evolutionary models of the thrifty phenotype, which refers to developmental plasticity, have been presented. These include (A) the weather forecast model of Bateson, (B) the maternal fitness model of Wells, (C) the intergenerational phenotypic inertia model of Kuzawa, and (D) the predictive adaptive response model of Gluckman and Hanson. These models are compared and contrasted, in order to assess their relative utility for understanding human ontogenetic development. The most broadly applicable model is model A, which proposes that developing organisms respond to cues of environmental quality, and that mismatches between this forecast and subsequent reality generate significant adverse effects in adult phenotype. The remaining models all address in greater detail what kind of information is provided by such a forecast. Whereas both models B and C emphasise the adaptive benefits of exploiting information about the past, encapsulated in maternal phenotype, model D assumes that the fetus uses cues about the present external environment to predict its probable adult environment. I argue that for humans, with a disproportionately long period between the closing of sensitive windows of plasticity and the attainment of reproductive maturity, backward-looking models B and C represent a better approach, and indicate that the developing offspring aligns itself with stable cues of maternal phenotype so as to match its energy demand with maternal capacity to supply. In contrast, the predictive adaptive response model D over-estimates the capacity of the offspring to predict the future, and also fails to address the long-term parent-offspring dynamics of human development. Differences between models have implications for the design of public health interventions.

## Introduction

For much of the 20th century, evolutionary biologists concentrated on gene-based models. Adaptation was assessed in terms of genetic fitness, evaluated in terms of the number of offspring produced by an organism and its relatives. By the end of the century, however, considerable attention was being devoted to within-lifetime adaptation of the organism, termed phenotypic plasticity (Schlicting and Pigliucci, 1998; West-Eberhard, 2003). Over the last two decades in particular, biologists and medical researchers have converged on a common issue from contrasting perspectives. While biologists have acquired evidence for the capacity of organisms to improve their fit with the environment during development, medical researchers have observed how certain early experiences appear to increase the risk of common human diseases.

An increasing volume of epidemiological research on humans supports the hypothesis that early-life experience predicts adult health status. In particular, the components of the metabolic syndrome (hypertension, type 2 diabetes and cardiovascular disease) are associated with growth patterns in fetal life, infancy and childhood, implicating nutrition as the underlying mechanism (Barker, 1998; Barker et al. 2002). The initial such findings contrasted strongly with the prevailing notion that adult diseases such as type 2 diabetes were primarily genetically determined.

To account for the association between low birthweight and increased later risk of type 2 diabetes, Hales and Barker (1992) proposed the “thrifty phenotype” hypothesis. This hypothesis focused on the ontogenetic development of the pancreas, and proposed that poor fetal growth compromised pancreatic function in the long-term. This effect would increase the future risk of developing type 2 diabetes, then considered an “adult-onset” disease, if the individual subsequently encountered a rich diet with high metabolic load. Subsequently, Hales and co-workers (Petry et al. 1997) suggested that other organs might likewise be compromised by early undernutrition, generating analogous risks for other health outcomes.

The thrifty phenotype hypothesis thus represents a generic model for the interaction between early experience and later outcome. The thrifty phenotype model is not the only one that has been proposed to explain associations between fetal size and later disease risk. The thrifty genotype model of Neel (1962, 1999) proposed that genetic factors could underlie risk of type 2 diabetes. Although evidence of thrifty genes, implicated both in low birth weight and increased risk of subsequent diabetes, has recently been obtained (Hattersley et al. 1998), these two models do not in fact conflict. The thrifty genotype model refers to long-term effects of selection on population genotype, whereas the thrifty phenotype refers to the capacity of all members of a species to experience plasticity during early development (Wells, 2003).

The thrifty phenotype hypothesis encapsulates a number of important concepts relating to ontogenetic development and long-term health. For medical researchers, the hypothesis highlights the possibility that improvements to adult health might depend on interventions early in life, independently of attempts to improve diet and activity levels during adulthood itself. For evolutionary biologists, the key issue concerns the way in which information is incorporated into phenotype during ontogenetic development, and whether this process is adaptive or not.

In the last few years, both evolutionary biologists and medical researchers have developed evolutionary models for the thrifty phenotype in humans. As will be seen below, these models offer different perspectives on a common theme, and emphasise different issues. It is therefore appropriate to consider their relative merits, and the extent to which they can be integrated. The aim of this review is to describe how each model addresses the incorporation of environmental information during development, and to assess their relative utility for understanding the epidemiology of human disease.

## Induction of Phenotype and the Programming of Disease

The association between early experience and later health status is itself controversial, due to the ambiguity of birthweight as an indicator of fetal growth. Whilst low birthweight infants could be said to have achieved lower rates of growth in utero, they also tend to experience faster “catch-up growth” in early postnatal life. This has led to debate concerning the relative importance of fetal versus postnatal growth for later health outcomes.

Animal studies have provided compelling evidence that undernutrition *in utero* influences the development of certain organs (Langley Evans, 2003). Lucas (1991) coined the term “programming” to describe the process whereby early experience impacts on the phenotype of the organism, and many authors within the biomedical community now refer to the “nutritional programming of adult diseases”. From a broader biological perspective Bateson (2001) has criticised the term “programming”, as it suggests that a given environmental cue contains “instructions” for diseases that will subsequently emerge. He has proposed the term “induction”, previously used in developmental biology to refer to a range of processes influencing the developing phenotype, and this article adopts this approach.

Despite strong evidence from animal studies for associations between fetal growth and later outcome, it is clear that postnatal growth rate is also implicated in the induction of adult phenotype, including in humans. In many epidemiological analyses, the association between human birth weight and adult disease risk emerges most strongly when adjustment is made for adult weight. It has been argued that the optimum interpretation of such statistics is that *change* in weight between birth and adult life is the best predictor of adult disease risk (Lucas et al. 1999). Certainly many data sets show an interaction between fetal growth and subsequent weight gain, with the strongest risk of disease associated with those born small who have caught up the most in postnatal life (Byrne and Phillips, 2000; Adair and Cole, 2002; Barker et al. 2002; Bavdekar et al. 1999).

Singhal and Lucas (2004) have proposed that postnatal growth alone comprises the key mechanism in the induction of disease. Nevertheless, their “growth acceleration” hypothesis ignores the fact that rapid post-natal growth is associated with prior growth retardation in most species (Metcalfe and Monaghan, 2001), and that “acceleration” in growth rate can only be defined in terms of prior growth trajectory. It is therefore inappropriate to separate out specific growth periods as the mechanism inducing deleterious outcome, rather it is only possible to compare the magnitude of effects attributable to interventions in different time periods. As yet, research in humans has generated evidence for the effects of nutritional intervention in the infant period (Singhal et al. 2001, 2003, 2004) that cannot be matched for fetal life, due to both ethical and physiological factors preventing analogous experiments during pregnancy.

A further difficulty in resolving the issue of whether prenatal or postnatal life is more important in the induction of human diseases derives from the fact that epidemiologists typically study morbidity or mortality as their outcome, whereas those conducting intervention trials tend to focus on physiological markers of risk. Hales and Barker (1992) for example suggested that poor fetal growth might compromise beta cell function, whereas Singhal and colleagues (2004) have linked rapid infant growth with a marker for insulin resistance. Since type 2 diabetes is now considered to be a “2-hit” disease, in which insulin resistance is necessarily accompanied by beta cell defects that prevent compensatory up regulation of insulin secretion (Bergman et al. 2002), it remains most plausible that both fetal and postnatal growth periods play important roles in the induction of this disease. A similar scenario is possible for other metabolic outcomes. However, the importance of the fetal period is demonstrated by the substantial loss of plasticity over the infant period, despite the persistence of growth over almost 2 decades post-conception (Wells, 2003). This discrepancy can be attributed to the fact that few of the rounds of cell division occur during postnatal life (Milner, 1989).

It is clear that humans can experience significant variability in fetal growth. While genetic factors clearly contribute to this variability, birthweight does not appear to be strongly influenced by genetic factors (Clausson et al. 2000; Baird et al. 2001; Magnus et al. 2001), and nutritional supply *in utero* is well-established as the primary factor influencing fetal growth. The thrifty phenotype model encapsulates the notion that fetal growth reflects not only placental nutrition, but also the acquisition of *information* about environmental quality. Whilst intuitively attractive, this notion requires critical appraisal, in order to elucidate the nature of such information.

Evolutionary perspectives on the thrifty phenotype must therefore address several related questions. First, how is information about environmental quality obtained? Second, how is this information used to guide organogenesis and ontogenetic development? Third, what exactly does the information consist of? Fourth, how reliable is the information that can be obtained? As will become clear below, these questions have been addressed by a range of evolutionary models.

### (A) The weather forecast model of Bateson

In their book “*Design for a life*”, Bateson and Martin (1999) discussed how the developing organism is capable of developing along a number of alternative pathways. The particular pathway achieved is associated with cues experienced by the organism during early development. Examples include the coat colour of voles reflecting cues of seasonality, and the growth rate of baboon males reflecting the social environment. These authors proposed that the developing organism obtains a “weather forecast” of the quality of the external environment, and uses this information to guide development. The organism may also subsequently select a niche for which it is well adapted.

In subsequent articles, Bateson (2001) and colleagues (Bateson et al. 2004) focused more specifically on how the human fetus receives cues of external environmental quality through its nutritional experience in utero. Whilst Hales and Barker (1992) had focused specifically on the altered insulin metabolism of low birthweight babies, Bateson emphasised a broader range of putative adaptations to a poor quality environment, including smaller body size and reduced metabolic rate (see Waterlow, 1999). Such traits could not be considered ideal, but might represent a strategy of “making the best of a bad job” (Bateson, 2001).

Although in some fast-maturing species (such as the freshwater crustacean, Daphnia, and small mammals such as rodents) the weather forecast obtained by the developing organism may prepare it appropriately for its adult environment, this issue is more complex in humans due to their lengthy developmental period. Bateson (2001) and colleagues (Bateson et al. 2004) suggest that the quality of the *childhood* environment, and its possible mismatch with experience *in utero*, may be particularly important for the risk of later disease. During World War II, many populations were exposed to famine in utero, and the resulting reduction in mean birth weight and length may be interpreted as an adaptive short-term response of fetuses to severe undernutrition, prioritising survival over optimal body size and composition. However, whether this early-life exposure was associated with adult disease was dependent on the quality of the environment during childhood. Low birth-weight Dutch babies who experienced catchup growth had a high risk of the metabolic syndrome in adulthood, whereas low birth-weight Russian babies who remained poorly nourished throughout childhood showed no such association (Stanner and Yudkin, 2001). The degree of match between childhood experience—the “conditions in which the child grows up” (Bateson, 2001)—and the weather forecast obtained during pregnancy is therefore extremely important, a point that will be discussed further below.

The notion that the developing organism responds to a weather forecast of the external environment, and is compromised when that forecast turns out to be incorrect, implies not only that thrifty phenotypes may be disadvantaged if they encounter improved environment in later life, but also that non-thrifty phenotypes may be disadvantaged if they experience poor environments in later life. Consistent with that hypothesis, Bateson (2001) noted anecdotal evidence that smaller men were more likely to survive imprisonment during the Second World War. He also observed an equivalent tendency for larger babies to have a higher risk of developing rickets on childhood exposure to famine in Ethiopia (Bateson et al. 2004).

Addressing the issue of the brief duration of periods of plasticity, Bateson (2001) has argued that plasticity cannot be maintained throughout development. Rather, as the developmental process continues, there is a loss of physiological capacity to alter phenotype and a given strategy must be consolidated. Other biologists have likewise considered the costs and benefits of plasticity (De Witt et al. 1998). At a certain cut-off point, the costs of altering phenotype must exceed any benefits attained. Thus critical windows close because for any given organ, a specific strategy in relation to structure and function must at a certain developmental time-point be adopted and maintained, resulting in humans in substantial loss in plasticity by the end of infancy.

The weather forecast model therefore represents a generalised version of the thrifty phenotype concept proposed by Hales and Barker (1992), linking it with evidence from many species that the developing organism responds to environmental cues during development. If these cues remain appropriate for environments encountered subsequently, the capacity of the developing organism to respond of them could be considered adaptive. Where the cues turned out to be mismatched with later environmental quality, the developmental strategy of the offspring would no longer be adaptive and in humans the risk of ill-health would be increased. However, Bateson and Martin (1999) also noted that different types of nutritional deficiency tend to invoke a common response in the phenotype. Thus placental dysfunction, which may occur regardless of external environmental conditions and hence conveys no useful information about environmental quality, invokes similar fetal responses to genuine maternal malnutrition.

To some extent all subsequent evolutionary models of the thrifty phenotype draw on the weather forecast model, which addresses the first two of the questions listed above—how is information obtained, and how is it used to guide development. What the other models have all focused in greater detail on is what kind of information enters the developing phenotype and whether it is reliable or not.

### (B) The maternal fitness model of Wells

Many areas of biology have been influenced by theoretical models of parent offspring conflict, first proposed by Trivers (1974) and subsequently elaborated by Haig (1992, 1993). These models focus on the theoretical argument that because a parent only shares 50% of its genes with each offspring, the two parties may be subject to a conflict of interest concerning the allocation of parental resources to offspring during the period of parental care. Briefly, parent-offspring conflict theory proposes that offspring fitness is maximised by a higher level of transfer of maternal resources than that which would maximise maternal fitness. Offspring demand and maternal supply are therefore proposed to be characterised by tension. In humans, the theory of parent offspring conflict has proven particularly valuable for interpreting the physiological interactions between mother and fetus during pregnancy (Haig, 1993).

The utility of such an approach has been questioned by Bateson (2004), who has suggested that maternal-fetal interactions could be considered as negotiations and exchanges of information rather than outright conflict. In fact the notion of a conflict of interest does not in any way negate the exchange of information—in any interaction, whether battle or cooperation, it is valuable for each party obtain information on the other’s intentions and resources. Equally, the fact that both mother and offspring clearly share interest in the successful rearing of that offspring need not negate the possibility of tension over the optimum supply of resources for doing so. This is discussed further below.

Medical researchers have provided a wealth of information about the way in which the fetus is provisioned via the placenta. The human placenta is haemochorial, a type that is relatively unusual in mammals, but common in old world primates. Haemochorial placentas are relatively aggressive and invasive of maternal uterine tissues (Ellison, 2001). This has been attributed to the high nutritional requirements of fetuses with large brains, a trait well known to be extreme in our species. In humans, the placenta requires both high maternal blood pressure and a high concentration of circulating nutrients in order to pass substrates to the fetus (Ellison, 2001). Maintaining such conditions involves a variety of components of maternal physiology.

Wells (2003, 2007) has used parent-offspring conflict theory to consider in more detail the kind of information that can be acquired by a human fetus via its weather forecast. Mammalian offspring developing *in utero* obtain no direct information on external environmental quality prior to birth, and instead receive only cues which have been transduced by maternal metabolism. On the one hand, this allows maternal metabolism to buffer short-term fluctuations in external environmental quality, and to provide a much more stable metabolic milieu for the vulnerable fetus. This of course is the primary function of maternal care. On the other hand, such capacity to buffer increases maternal control over offspring developmental trajectory.

In many species, such maternal control may still result in an allocation of resources that matches very closely with the interests of the offspring. In birds, for example, maternal phenotype influences egg composition such that many aspects of offspring phenotype (including gender, energy reserves, and immunocompetence) are well-fitted to the likely environment post-hatching. Such transmission of phenotype is known as a “maternal effect” (Mousseau and Fox, 1998), and represents the use of maternal information about environmental quality to improve the mother’s own reproductive fitness. As maternal fitness is maximised by investing directly in offspring fitness, there is a close fit between offspring needs and maternal supply.

Humans however are unusual in their extended period of growth, and in the effects of this growth pattern on the simultaneous maternal care of competing offspring. In most animal species, offspring become fully independent of the parents at the time of weaning, allowing the mother to devote her complete budget of resources to a single litter at any one time. In humans, in contrast, offspring only achieve physiological independence from the mother at a time of weaning, and remain dependent on maternal resources for food supplies for many years subsequently. Amongst the Ache, a foraging population from Paraguay, offspring only became fully independent of their parents in terms of energy supply at around 18 years of age (Hill and Hurtado, 1996). More generally, human children in foraging societies contribute only a minority of their dietary energy (Hewlett and Lamb, 2005).

The early growth patterns of humans therefore have major long-term implications for the maternal energy budget (Wells, 2003, 2007). This is particularly the case because critical windows during which nutrition regulates growth and size close by the end of infancy. After this period, growth is genetically canalised and has reduced (though not zero) sensitivity to nutritional conditions. For example, a high plane of nutrition in childhood may increase the rate of maturation (both growth rate and progression through puberty) (Parent et al. 2003), but has a relatively small effect on final size. Conversely, a low plane of nutrition temporarily slows growth, but subsequent catch-up to the original trajectory is attained when conditions improve. A recent analysis re-emphasised the implications of offspring growth rate for the human maternal energy budget, with Gurven and Walker (2006) arguing that the “human pattern of slow offspring growth between ages at weaning and puberty helps defer rising demand on parents with multiple, overlapping dependents”. Because early variability in size, reflecting fetal and infant nutrition, is preserved in offspring phenotype over the long term, it benefits the mother to regulate such early growth patterns in relation to her own phenotype.

The maternal fitness approach therefore proposes that the information received by the offspring in utero is not an accurate guide of the quality of the external environment. Human conception appears to be controlled by a mechanism that is sensitive to whether energy stores and energy flux rate are conducive to supplying the energy required for a pregnancy (Ellison, 2001; Wells, 2003). Once this mechanism has been activated, maternal metabolism has been shown to have extraordinary capacity to buffer subsequent environmental perturbations. Extreme famine conditions can reduce mean birth-weight by up to half a kilogram (Stanner and Yudkin, 2001; Stein et al. 2004) (still only a 15% reduction in mean birthweight), but in general human fetal development is remarkably robust to fluctuations in maternal energy supply. Thus the first conclusion of the maternal fitness approach is that the fetus is inherently protected from experiencing the poorest conditions, and hence never receives accurate cues of external famine.

On the other hand, the mother’s traits that regulate the supply of energy to the fetus are strongly influenced by her own development and her nutritional status prior to conception (Wells, 2003, 2007). Nutritional intake during pregnancy has been shown in numerous studies to have less influence on offspring birthweight than maternal nutritional status prior to pregnancy or maternal birthweight. Supplementation studies have relatively little effect on mean birth weight even if the mother is seriously malnourished, while maternal obesity is associated with relatively small increases in birth weight (Wells, 2007). Thus the second conclusion of the maternal fitness approach is that even when the external conditions experienced during pregnancy are extremely high-quality, the fetus cannot experience this directly either, and hence never receives accurate cues of external plenty. Rather, very large fetuses only occur when maternal metabolic control is deranged, as in maternal gestational diabetes (Carrapato, 2003; Catalano et al. 2003).

Thus the maternal fitness approach emphasises that the information passing to the offspring does not represent accurate cues of current external environmental quality, but rather comprises a summary of maternal phenotype that reflects the mother’s own development. This information is nevertheless of high value to the offspring because it allows its demand for energy to be aligned with long-term maternal capacity to supply. Clearly this strategy is ideal for a species in which the offspring remains dependent on maternal energy allocation for many years following birth. Put another way, the aspect of the environment that is of most importance to the human offspring is its mother, given her long-term influence under “natural conditions” on the offspring’s energy supply. According to this approach, the weather forecast provides information about environmental quality *in the past*, and more specifically about how cumulative past environments impacted on maternal development. The offspring could certainly be said to respond adaptively to these cues, but nevertheless to be obliged to submit to maternal reproductive strategy in doing so (Wells, 2003).

It has been suggested (by an anonymous reviewer) that the maternal fitness model is flawed, because it could never benefit a mother to produce offspring with the thrifty phenotype. This view is mistaken because it fails to recognise the benefits to *both* parties of offspring demand being matched to maternal capacity to supply. As reviewed comprehensively by Haig (1993), the transfer of nutrients to the offspring *in utero* is achieved through a system under tension, through the action of fetal and maternal hormones. Such physiological tension continues during lactation, but after weaning any tension must be shifted to the arena of behaviour. Long-term behavioural tension between mother and offspring is detrimental to both parties, wasting resources and possibly attracting predators. The use of brief sensitive windows during pregnancy and lactation to align offspring growth trajectory with maternal phenotype therefore represents a valuable negotiation. The growth trajectory that that has manifested by the time of weaning remains set in offspring phenotype, when the critical windows close (Wells, 2003).

The importance of maternal phenotype for offspring growth trajectory is also important in accounting for the association between early growth and risk of disease. According to the maternal fitness model, the risk of disease is increased when the effect of maternal phenotype on childhood development is diminished, such that the alignment between the parties is disrupted (Wells, 2007). During human evolutionary history, the importance of the mother in allocating resources to her offspring would have engineered consistency in energy availability to each offspring throughout its infancy and childhood. In industrialised populations, in contrast, maternal physiology only impacts briefly on the offspring during pregnancy, and in some cases lactation (typically of brief duration compared to non-industrialised population). After weaning, the impact of maternal physiology on offspring growth tends to be negated and economic conditions, rather than maternal nutritional status, determine dietary quality. The low birth-weight baby is likely to be aggressively fed during infancy, and is likely to encounter a rich diet throughout childhood. Thus a disparity between the long-term maternal phenotype and childhood dietary quality is considered fundamental to the aetiology of the metabolic syndrome (Wells, 2007), supporting the emphasis of Bateson and colleagues (2004) on the conditions in which the child grows up. Consistent with this approach, Barker and colleagues have now identified childhood growth patterns as a key factor mediating the association between low birthweight and adult disease (Bhargava et al. 2004; Barker et al. 2005).

### (C) The intergenerational phenotypic inertia model of Kuzawa

An alternative perspective on the type of information conveyed by fetal nutrition was developed by Kuzawa (2005). As emphasised in the maternal fitness perspective, Kuzawa noted that short term fluctuations in maternal energy supply are not passed on to the fetus, and that variability in birth weight of the offspring more closely reflects longer-term components of maternal phenotype, and indeed phenotype of the grandmother. Kuzawa has therefore argued that intrauterine nutrition offers an integrated signal of longer-term nutritional history, not only of the mother but also of her recent ancestors, and that this signal acts as means of damping short-term noise to provide a more coherent index of ecological conditions. He has termed this “intergenerational phenotypic inertia”, and has focused in particular on the consistency and reliability of signals that are obtained during the early life weather forecast.

To elucidate further the nature of information received by the human fetus, Kuzawa (2007) has compared the biology of humans with that of voles. For example, the Montane vole (Microtus montanus) inhabits a highly seasonal environment, but with a lifespan of one year or less the conditions experienced by any mother during pregnancy do not represent an accurate forecast of the conditions to be encountered by the offspring. Kuzawa has noted two mechanisms by which the fetal vole can prepare itself for its postnatal environment. First, information about external season is conveyed by concentrations of the hormone melatonin, produced by the mother’s pineal gland in response to day-length and transmitted across the placenta (Horton, 1984). This mechanism allows the vole to identify which period in the predictable seasonal cycle it will be born into. Second, information about the relative abundance of grasses, on which the fetal vole will feed after birth, is conveyed by the concentration of maternal metabolites likewise crossing the placenta (Berger and Negus, 1992). This second mechanism allows the fetal vole to adopt a growth trajectory compatible with external food supplies. Collectively, these mechanisms represent what Kuzawa (2007) has termed forward-looking predictive adaptive plasticity.

In contrast to the vole, human development is best considered to comprise a backward-looking version of plasticity. Kuzawa (2007) notes that the problem facing humans is the exact opposite of that faced by voles. The human fetus, destined to experience many seasons in postnatal life, should not target adaptation disproportionately at the current external environment, but should rather align itself with longer-term signals of ecological conditions. Such signals derive from maternal and grand maternal phenotype, summing up recent nutritional experiences of the matriline.

Such a mechanism offers the capacity to filter out transient environmental fluctuations and to track instead consistent long-term trends. Although this mechanism inevitably involves a time lag, Kuzawa (2005) has suggested that intergenerational phenotypic inertia provides an intermediary timescale of adaptive response, faster than genetic change but slower than simple plasticity within individual development. In other words, the mechanism offers the means of benefiting from plasticity without succumbing to inadvertent tracking of very transient environmental qualities.

The notion that intergenerational plasticity represents an adaptive mechanism intermediary between the level of genes and physiology is supported by increasing evidence for the contribution of epigenetic transmission to the induction of human phenotype. Epigenetic transmission refers to alterations in the methylation of DNA, which influences gene expression without altering gene content per se. Kuzawa (2007) notes that different traits appear to have different durations of epigenetic transmission, equivalent to variability in the strength of effect exerted by matrilineal experience on subsequent generations, and that the impact of offspring genotype on induced aspects of the phenotype allows a link between ancestral experience rather than current experience with offspring genotype. Thus even this genetic component of the organism development is mediated by ancestral experience.

Kuzawa has also suggested (personal communication) that it would benefit the offspring to close critical windows at the time of weaning simply because information about environmental quality obtained directly from the childhood of that is no longer averaged by maternal physiology, and hence is a less reliable guide for developmental trajectory. This suggestion is consistent with the maternal fitness approach, however I have argued that because the offspring is therefore obliged to commit to its growth trajectory so early in development, the mother is able to exploit the period of physiological parental care to *manipulate* that offspring trajectory (Wells, 2003).

Finally, Kuzawa (2007) has also noted that human developmental plasticity may owe its current form not only to its beneficial capacity for adaptation, but also to its persistence from early vestigial forms which, comprising an integral component of ontogeny, cannot be excised from the developmental process. This point is further discussed below, and is of importance because it highlights the fact that a single broad mechanism of plasticity can be exploited and adapted in different ways in different types of organism.

Kuzawa’s model shares many similarities with the maternal fitness model. Both models focus on the use of information about the past, encapsulated in maternal phenotype, to guide offspring development. However, whereas Wells (2003, 2007) has emphasised the manipulative influence of maternally-derived information, Kuzawa (2005, 2007) has emphasised its reliability as a guide for offspring growth strategy. These perspectives are essentially complementary and extend the weather forecast model by elucidating the nature of information entering offspring phenotype.

### (D) The predictive adaptive response model of Gluckman and Hanson

Gluckman and Hanson have focused in more detail on the notion that early plastic responses by the offspring represent make long-term predictions of environmental quality. In their book “The fetal matrix” (Gluckman and Hanson, 2004a) and a succession of related review articles (Gluckman and Hanson, 2004b, 2007; Gluckman et al. 2005a; 2005b), they suggest that the fetus constantly “interprets” the environment created by the maternal metabolic milieu and placental function. “Nutrients passing into the fetal blood from the placenta act as vital coded messages about the external environment, which the struggling fetus utilises to make strategic adaptive choices” (Gluckman and Hanson, 2004a: 61). Using such cues, these authors argue, the fetus predicts its likely future environment and adopts an appropriate developmental trajectory.

Like Kuzawa (2005, 2007); Gluckman and Hanson draw inferences from apparent common features in the biology of humans and voles. Unlike Kuzawa, however, they pay less attention to possible differences between genera in the exact information being transmitted to offspring phenotype. This leads them to considering both genera as utilising a common mechanism, that of using cues obtained during pregnancy in order to adjust phenotype of the *mature* organism. Coining the term “predictive adaptive responses”, they argue that some elements of plasticity explicitly do not confer immediate advantage but induce phenotype in the expectation of a long-term future predicted environment, specifically that where reproduction will occur (Gluckman and Hanson, 2004; Guckman et al. 2007). This hypothesis may have validity in short-lived species such as rodents, but it generates a number of problems in the context of humans. Problematic issues include the reliability of cues over long time periods, and the transfer of information between individuals subject to conflicts of interest.

Conflict of interest is inevitable when information passes between two individuals that are not genetically identical. Using the concept of the “extended phenotype” Dawkins (1982) has previously considered all behaviour as “genetic action at a distance”, hence the information that derives from maternal physiology inevitably comprises maternal genetic action on, and manipulation of, offspring phenotype. Haig (1996) has likewise stated that all information must be considered as manipulation, since any behaviour that fails to manipulate (i.e. exert an effect) could be said to contain no information. Understanding the manipulatory role of information transfer during pregnancy is therefore essential for clarifying what adaptations the offspring can actually make.

In the examples of rodents considered by both Kuzawa and Gluckman and colleagues, the plasticity of the offspring is quite limited. Offspring physiology generates a relatively fixed (i.e. blind) response to given cues, and maternal phenotype (in turn reflecting current ecological conditions) is able to provide such cues with a good degree of reliability. Gluckman and colleagues’ approach thus departs from the conventional parental effects model by conferring on the offspring a more proactive role in evaluating the current environment and predicting the future environment.

Such proactive prediction of the future environment by the offspring is however particularly implausible in long-lived humans. Hominin evolution consistently occurred in relatively unstable environments—indeed recent hominin biology has been strongly influenced by the processes of colonisation and niche construction, resulting for example in rapid depletion of food supplies through over-exploitation (Diamond, 1998). Such characteristics suggest that current ecological conditions could only fortuitously offer an accurate prediction of those likely to be encountered in two decades time. Similarly, Gluckman and colleagues have ignored the extraordinary capacity of human maternal metabolism to buffer ecological fluctuations during pregnancy. Far from placental nutrition acting as “vital coded messages about the external environment”, the fetus is substantially protected from directly experiencing the external environment and instead receives, as both Wells (2003) and Kuzawa (2005) have emphasised, information about matrilinial ancestral environments.

It is certainly true that there are elements of human physiology which respond adaptively to environmental cues during fetal life. For example, human sweat gland number appears strongly influenced by thermal load experienced in early life (Kawahata and Sakamoto, 1951) and represents early adaptation, given that geographical variation in the climate is broadly consistent from year to year. However, precisely because of such likely consistency throughout the organism’s lifetime, fetal prediction of post-natal thermal environment does not represent a predictive adaptive response according to the definition of Gluckman and Hanson (which refers specifically to adaptations targeted at the long-term future rather than the present). The timing of puberty also varies in relation to environmental conditions (Gluckman and Hanson, 2006), but again is sensitive to the rate of childhood weight gain. Hence despite an association between low birth weight and earlier puberty this trait likewise cannot be considered primarily as a predictive adaptive response.

Gluckman and colleagues (2007) have discounted the argument that variability in offspring phenotype benefits maternal fitness, by arguing that one of the “hallmarks of offspring thriftiness”, insulin resistance, does not develop until “well after birth” in small-for-gestational-age individuals, and hence could not benefit the mother. In fact, recent data show that small-for-gestational-age infants show differences in insulin secretion from birth onwards, and manifest differences in insulin resistance from around one year of age onwards (Soto et al. 2003; Mericq al, 2005). Improved insulin secretion appears to promote length gain during the first year of life (Soto et al. 2003). However, where that length gain is accompanied by a high level of weight gain, increased fasting insulin levels also emerge, implying insulin resistance (Mericq et al. 2005). Between two and four years of age this insulin resistance then promotes central adiposity (Ibáñez et al. 2006). This growth profile suggests that the metabolism of small-for-gestational age infants is “set up” so as to recover early deficits in length, during the period of lactation. Under the “natural conditions” of lactation, catch-up growth in weight would be constrained by maternal breast-milk supply, limiting the development of insulin resistance and abdominal adiposity in the offspring. In modern Western in environments, however, such constraints on catch-up growth can be released (e.g. by formula-feeding, or rich childhood diet), whereupon insulin resistance increases. Thus, contrary to the argument of Gluckman and colleagues (2007), insulin metabolism in early life appears closely attuned to maternal offspring dynamics, and indeed it is much more difficult for their model to explain why rapid infant weight gain (apparently indicating improved environmental conditions) should paradoxically promote insulin resistance and “thriftiness” over the longer term. Instead, the small-for-gestational age infant appears well adapted to its *early-life* environment, in other words its *mother’s* phenotype.

Gluckman and Hanson (2007) also discount the importance of maternal fitness by highlighting the persistence of induced effects across a number of generations. According to their argument, this must imply predictive adaptation not only of the offspring but also of its descendants to long-term future environments, which these authors consider of particular importance in human evolution. Once again however their emphasis on forward-looking as opposed to backward-looking models is flawed. Instead of considering that the current offspring will benefit by pre-emptively influencing its own grand-offspring, a more appropriate model is that the current offspring is unable fully to shake off environmental effects that acted on its grandparents. The long-term effects of matrilineal experience on subsequent generations is simply indicative of the high sensitivity of hominin female biology to the ecological environment, obliging females to constrain investment in their offspring if their own resources (encapsulated in their size, nutritional status and developmental history) are poor.

Finally, Gluckman and colleagues (2007) argue that similarities in the “survival phenotype” across species imply a common adaptive mechanism in relating early life of plasticity to lifetime reproductive fitness. That human offspring, as in other species, make integrated responses to cues of nutritional supply is not disputed, and Gluckman and colleagues are correct to note that natural selection appears to have favoured a broadly similar response to low nutrient intake in a range of species. Where they are mistaken however is in assuming that this response is, in all species, a universal mechanism for preparing infant organisms for their *adult* environment. Kuzawa (2007) noted that the thrifty phenotype derives in part from vestigial components of physiology that cannot be excised from the developmental process. This is consistent with the argument of Bonner (1965) that it is largely through re-ordering of the life cycle and growth process that different species evolve. During such re-ordering, developmental plasticity may itself be exploited in novel ways. As discussed above, human growth is extremely unusual in its lengthy duration, and yet organogenesis is largely completed during infancy. The extending and reordering of human growth (Bogin, 2001) may thus be considered to have involved some changes in the functions of plasticity itself, including the canalisation of growth from infancy onwards so as to remove it from nutritional regulation for the majority of the growth period (Wells, 2003). It was Francois Jacob (1977) who famously referred to evolution as a process of “tinkering”. Though both humans and voles benefit from developmental plasticity, the use of common physiological systems in their contrasting processes of induction may be considered a product of such tinkering.

## Discussion

Different models of the thrifty phenotype emphasise different dimensions of a broadly adaptive process. All the models incorporate the notion of the developing organism obtaining information via maternal nutrition as some kind of forecast concerning environmental conditions, as proposed by Bateson and colleagues (1999, 2001, 2004). The main area of disagreement comprises what information is contained in this forecast. Both Wells (2003, 2007) and Kuzawa (2005, 2007) have emphasised that information by human offspring pertains much more to the recent past than to current ecological conditions, and therefore describes what the *mother* is like and has experienced, rather than what *external* physical conditions are like. In contrast, Gluckman and Hanson (2004a, 2004b) suggest that the fetus receive cues concerning current external conditions, and uses these to predict its adult environment.

Differences between the models of Kuzawa and Wells are more subtle, and represent differential emphasis on two complementary aspects of the process. Kuzawa’s model focuses on the reliability of maternal phenotype as a signal of broader ecological trends, and considers the value of adopting a developmental trajectory that reflects recent ancestral experience rather than current transient conditions. Wells’ model focuses on the submission of offspring developmental trajectory to maternal control, deriving from a trade-off between maternal and offspring fitness. Both models therefore emphasise the way in which offspring growth trajectory is aligned with maternal phenotype, and both perspectives are valuable. Given the long-term contribution of the mother to offspring energy supply, such alignment matches offspring demand to maternal capacity to supply, which in turn represents a multigenerational tracking of the relationship between ecological quality and maternal capacity to provision.

The merits of competing models should ideally be tested using data. Three different approaches are relevant in this context. First, mathematical models based on game theory may be used to simulate associations between optimum strategies across the range of environmental conditions, and to identify the impact of differing approaches to the incorporation of information in such strategies.

Second, several scenarios provide human data highly relevant for comparing between models. Twins discordant for birthweight, and successive offspring of the same parents born at different times of the year in highly seasonal environments, represents a rich source of information for assessing the relative importance of current external conditions versus maternal phenotype for inducing offspring phenotype. For example, data from the Gambia failed to demonstrate any association between season of birth and metabolic outcome in childhood (Moore et al. 2001).

Third, improved understanding of the role of information transmission in the induction of offspring phenotype would derive from rigorous examination of the archaeological record, clarifying the nature of environmental fluctuations over the last 2 million years including their magnitude, frequency, and consistency. The relative value of responding to external ecological conditions depends very much on whether signals obtained over a short time period prove reliable subsequently. Some aspects of the environment may change relatively slowly, such as average environmental temperature. Other aspects may change on a much more rapid timescale, with for example seasonal or annual variability in the availability of prey animals, or key vegetable resources.

Each of these approaches offers a variety of opportunities for explicitly challenging the different models, and identifying the most robust.

## Conclusion

The thrifty phenotype hypothesis was originally developed in order to improve understanding of the association between early experience and later risk of disease. All models are consistent in suggesting that disease risk is increased if there is disparity between experience *in utero* and subsequent environmental conditions. Bateson (2001) emphasised the importance of this disparity occurring during the period of childhood growth, and this perspective is supported by Wells’s maternal fitness model suggesting that disease risk is increased when the consistent influence of maternal phenotype on offspring childhood growth is minimised (Wells, 2007). Kuzawa (2005) and Gluckman and Hanson (2004a, 2004b) have focused more on disparity between adult lifestyle and fetal experience. It is likely that any disparity significantly increases the risk of disease, and adult obesity certainly generates adverse impacts on health, including elevated risk of the metabolic syndrome. Nevertheless, recent studies examining the role of growth patterns in the aetiology of heart disease and type 2 diabetes (Bhargava et al. 2004; Barker et al. 2005) support the notion that excess childhood weight gain is of particular importance. Another study, demonstrating increased risk of obesity in relation to rapid weight gain from formula-feeding merely in the first 8 days of life (Stettler et al. 2005), also highlights the deleterious effect of over-riding maternal physiology.

The thrifty phenotype hypothesis has proven extremely valuable in elucidating the aetiology of the metabolic syndrome. Evolutionary models contribute by clarifying the pathways by which information enters the developing organism and guides ontogenetic trajectory. Such models are not just of academic interest, rather identification of the most accurate model will aid target appropriate interventions to improve population health. The model of Gluckman and colleagues emphasises fetal life as the life-course period key to the induction of adult ill-health, whereas other models emphasise broader disparity between maternal metabolic profile and offspring developmental experience. Further data collection will enable these alternatively models to be tested more thoroughly.
